# Supplementary material for: What does it take to learn the rules of RNA base pairing? A lot less than you may think
Source: bioRxiv. 2025 Aug 2:2025.07.31.668042. Preprint. [Version 1] doi: 10.1101/2025.07.31.668042 (PMC12324431; doi:10.1101/2025.07.31.668042)
Supplement: Supplement 1 [file media-1.gz › supplemental_material/src/rscape_v2.6.0.tar.gz!/rscape_v2.6.0/lib/hmmer/documentation/userguide/inclusions/alidisplay-serialize.pdf]

<table>
<tr><td>Size (4 bytes)</td><td>N (4 bytes)</td><td>Hmmfrom (4 bytes)</td><td>Hmmto (4 bytes)</td></tr>
<tr><td>M (4 bytes)</td><td colspan="2">Sqfrom (8 Bytes)</td><td></td></tr>
<tr><td colspan="2">Sqto (8 Bytes)</td><td colspan="2">L (8 Bytes)</td></tr>
<tr><td colspan="2">String Presence Flags (1 Byte)</td><td colspan="2"></td></tr>
<tr><td colspan="4">Rfline String (N+1 bytes or 0)</td></tr>
<tr><td colspan="4">Mmline String (N+1 bytes or 0)</td></tr>
<tr><td colspan="4">Csline String (N+1 bytes or 0)</td></tr>
<tr><td colspan="4">Model String (N+1 bytes)</td></tr>
<tr><td colspan="4">Mline String (N+1 bytes)</td></tr>
<tr><td colspan="4">Aseq String (N+1 bytes or 0)</td></tr>
<tr><td colspan="4">Ntseq String (3N+1 bytes or 0)</td></tr>
<tr><td colspan="4">Ppline String (N+1 bytes or 0)</td></tr>
<tr><td colspan="4">Hmmname String (Variable Length)</td></tr>
<tr><td colspan="4">Hmmacc String (Variable Length)</td></tr>
<tr><td colspan="4">Hmmdesc String (Variable Length)</td></tr>
<tr><td colspan="4">Sqname String (Variable Length)</td></tr>
<tr><td colspan="4">Sqacc String (Variable Length)</td></tr>
<tr><td colspan="4">Sqdesc String (Variable Length)</td></tr>
</table>
